# Single image de-raining by multi-scale Fourier Transform network

**DOI:** 10.1371/journal.pone.0315146

**Published:** 2025-03-18

**Authors:** Chaobing Zheng, Yao Yao, Wenjian Ying, Shiqian Wu

**Affiliations:** 1 Institute of Robotics and Intelligent Systems, School of Information Science and Engineering, Wuhan University of Science and Technology, Wuhan, China; 2 Division of Mechanical Manufacturing and Intelligent Transportation, Beijing Institute of Metrology, Beijing, China; 3 College of Weapon, Naval University of Engineering, Wuhan, China; Beijing University of Technology, CHINA

## Abstract

Removing rain streaks from a single image presents a significant challenge due to the spatial variability of the streaks within the rainy image. While data-driven rain removal algorithms have shown promising results, they remain constrained by issues such as heavy reliance on large datasets and limited interpretability. In this paper, we propose a novel approach for single-image de-raining that is guided by Fourier Transform prior knowledge. Our method utilises inherent frequency domain information to efficiently reduce rain streaks and restore image clarity. Initially, the rainy image is decomposed into its amplitude and phase components using the Fourier Transform, where rain streaks predominantly affect the amplitude component. Following this, data-driven algorithms are employed separately to process the amplitude and phase components. Enhanced features are then reconstructed using the inverse Fourier Transform, resulting in improved clarity. Finally, a multi-scale neural network incorporating attention mechanisms at different scales is applied to further refine the processed features, enhancing the robustness of the algorithm. Experimental results demonstrate that our proposed method significantly outperforms existing state-of-the-art approaches, both in qualitative and quantitative evaluations. This innovative strategy effectively combines the strengths of Fourier Transform and data-driven techniques, offering a more interpretable and efficient solution for single-image de-raining (Code: https://github.com/zhengchaobing/DeRain).

## 1 Introduction

Adverse conditions, low light [1–5], haze [6–9], particularly rain [10], can severely degrade image quality, blurring critical details and directly impacting the stability of image processing systems. The removal of rain from images is essential for preserving visual clarity and maintaining information integrity across various applications, such as surveillance, autonomous vehicles, and smart transportation [10–14]. Raindrops on camera lenses or in the scene itself introduce streaks and distortions that obscure important information, impairing the performance of computer vision algorithms tasked with object detection [15], tracking [16, 17], and scene understanding [18]. Mitigating these adverse effects through image rain removal techniques is crucial to enhancing the reliability and efficiency of these systems, enabling their seamless operation even under challenging weather conditions. Furthermore, in specialised fields like intelligent traffic monitoring, remote sensing, and meteorological analysis, the ability to accurately interpret visual data during rainy conditions is indispensable for precise decision-making and data-driven interventions. For instance, in autonomous vehicles, the presence of rain-induced artefacts can significantly compromise the accuracy of obstacle detection and navigation, posing safety risks. Similarly, in remote sensing applications, where images are used for environmental monitoring and urban planning, the integrity of the visual data is paramount.

The development and refinement of image rain removal methods are thus fundamental to ensuring the consistent functionality and performance of a wide range of visual systems in adverse weather. These techniques not only improve the visual output by restoring image clarity but also bolster the overall resilience and robustness of systems dependent on accurate visual information. By addressing rain-induced distortions, these methods contribute to the enhanced stability of applications where clear visual input is a prerequisite for effective operation [19].

Given the critical importance of rain removal in outdoor vision systems, the task of restoring rain-affected images has attracted significant attention from researchers. Methods for addressing this issue can be broadly classified into two categories [20, 21]: video-based approaches and single-image-based techniques. Single-image de-raining poses a greater challenge compared to video-based methods, primarily due to the limited information contained in a single frame. In video sequences, the availability of temporal data offers a distinct advantage, as multiple frames provide supplementary information that facilitates the identification and removal of rain streaks. Video-based algorithms can leverage the consistency of rain patterns across consecutive frames, making it easier to distinguish transient rain artefacts from static scene details. Moreover, rain-free frames in a video can serve as reliable references for reconstructing the scene, further aiding in rain removal efforts.

In contrast, the absence of temporal information in single-image de-raining makes it significantly more difficult to separate rain streaks from other scene elements. Rain streaks vary widely in terms of size, shape, orientation, and intensity, and they often overlap with important objects or textures within the image, complicating their removal. Without the benefit of a frame sequence, accurately reconstructing the rain-free scene becomes a formidable task. The single image must convey sufficient information for algorithms to effectively detect and eliminate rain while preserving essential image details. Consequently, researchers have been increasingly focused on developing sophisticated techniques to tackle the inherent complexities of single-image rain removal, devising methods that aim to isolate and eliminate rain streaks without compromising the structural integrity and visual fidelity of the underlying image content [22, 23]. Addressing this challenge requires the development of innovative solutions capable of capturing both local and global image features. These methods often rely on a combination of frequency domain analysis, attention mechanisms, and multi-scale neural networks to enhance their ability to differentiate rain artefacts from other high-frequency components. As a result, the field has witnessed the emergence of various advanced techniques designed to achieve accurate rain removal in the absence of temporal or supplementary information, contributing to the ongoing progress in single-image restoration tasks.

Existing single-image de-raining methods can be broadly categorised into two groups: physics-based methods [24–27] and data-driven methods [28–32]. While these algorithms often assume a uniform distribution of raindrops, real weather conditions are notably intricate, involving raindrops of diverse sizes and shapes, as well as factors such as wind and fog. Model-based methods, which fall under physics-based approaches, frequently rely on assumptions regarding the distribution and shape of raindrops. However, these assumptions may not hold true under complex weather conditions, presenting challenges in effectively removing raindrops in certain situations. Conversely, data-driven methods, while effective, often lack interpretability in their internal decision-making processes. Understanding why certain decisions are made can be challenging, thus limiting the transparency of these algorithms. Additionally, data-driven de-raining algorithms are highly sensitive to the data on which they are trained. Consequently, their de-raining performance can vary significantly across different domains. Some algorithms may excel in specific scenarios but struggle to generalise effectively to diverse situations, thereby limiting their overall performance and applicability.

This paper introduces an innovative algorithm for the removal of rain from images by seamlessly integrating data-driven and physics-based approaches. The inspiration for this hybrid method is rooted in a well-established property of the Fourier transformation: the Fourier phase spectrum preserves high-level semantics, while the amplitude spectrum encapsulates low-level features [33].

*Frequency Domain Analysis*: The Fourier Transform plays a crucial role in converting an image from the spatial domain to the frequency domain, offering deeper insights into the various frequency components that constitute the image. This transformation allows for the separation and examination of different patterns and structures within the image, which can be difficult to distinguish in the spatial domain alone. In the context of image de-raining, the Fourier Transform is particularly valuable as it enables the identification and analysis of the spectral signatures introduced by raindrops. Rain streaks, often appearing as high-frequency noise, manifest as distinct frequency components in the Fourier domain. By isolating these components, we can more effectively attenuate the influence of rain streaks, while preserving the underlying image details. This approach not only enhances the clarity of the restored image but also allows for more precise control over the removal process, improving the overall robustness of image de-raining algorithms. The frequency domain perspective thus offers a complementary technique to spatial domain methods, enabling a more comprehensive and efficient approach to rain removal.

**Fig 1:**
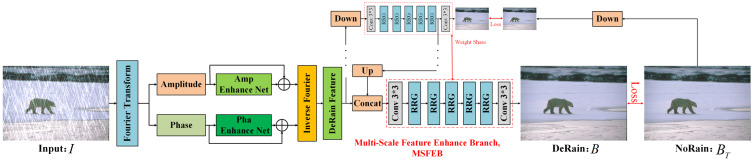
The proposed framework for single-image rain removal. *I* is a rainy image, *B* is a restored image, and $B_{T}$ is the ground truth image of *B.*

*Phase and Amplitude Information*: The Fourier Transform decomposes an image into two distinct components: phase and amplitude. In the context of image de-raining, phase information typically encodes high-level semantic details, such as the structure and spatial relationships within the scene, while amplitude captures low-level details, including variations in intensity and texture. This distinction allows for a more targeted approach in addressing the impact of rain streaks on both dimensions of the image. As illustrated in Fig 1, applying the Fourier transform separates the rainy image into its amplitude and phase components. Rain streaks predominantly affect the amplitude, where the high-frequency noise introduced by rain is most concentrated, whereas the phase component preserves the essential structural features of the background. Given these characteristics, it is intuitive and advantageous to handle amplitude and phase information independently in single-image rain removal tasks. Processing the amplitude allows for the removal of rain artefacts, while maintaining the integrity of the phase ensures that the underlying scene structure remains intact. By treating these components separately, the proposed method is better equipped to restore a rain-free image with greater clarity and precision.

To improve the algorithm’s capacity for handling dense raindrops, we introduce an additional refinement step for the rain-removed feature maps. This refinement process further enhances the visual quality of the de-rained images by ensuring that residual rain streaks are effectively suppressed. Our proposed algorithm integrates deep learning techniques with a physics-based prior model, creating a synergistic framework where deep learning enhances the robustness and adaptability of the system, while the prior model provides greater interpretability by leveraging established physical principles. This integration allows the complementary strengths of both approaches to be utilised, with deep learning excelling in extracting complex patterns and the prior model guiding the algorithm to ensure consistency with natural image statistics. The combination not only improves the overall accuracy but also provides a more interpretable and explainable solution, addressing common criticisms of purely data-driven models. As demonstrated in our experimental evaluations, the proposed de-raining algorithm consistently outperforms several state-of-the-art methods, both in terms of visual quality and quantitative metrics. These results underscore the advantages of combining deep learning with physics-based approaches, proving that such a hybrid framework offers substantial improvements in both performance and generalisation capacity compared to conventional methods [34].

The remainder of this paper is organised as follows. Section 2 provides an overview of related work, discussing the key advancements and limitations in existing single-image de-raining methods. In Section 3, we present a detailed explanation of our proposed de-raining algorithm, outlining its theoretical foundation and implementation framework. Section 4 showcases the experimental results, where we validate the effectiveness of the proposed method through both qualitative and quantitative analyses on various benchmark datasets. Finally, Section 5 summarises our contributions and offers insights into potential future research directions, highlighting the broader implications of our findings.

## 2 Relevant works

Rain removal has garnered significant attention from researchers aiming to address the challenge of restoring images affected by rain. These methods can be broadly classified into two categories: physics-based approaches and data-driven approaches.

*Physics-based methods*: Physics-based algorithms are fundamentally rooted in a thorough understanding and modelling of the physical processes related to raindrops and precipitation. This grounding allows them to effectively capture the characteristics and behaviour of raindrops, leading to greater interpretability. Users can thus gain insights into the mechanisms involved in the de-raining process, making these algorithms advantageous for applications where understanding the underlying processes is crucial. However, physics-based algorithms often rely on certain assumptions regarding factors such as the distribution, size, and shape of raindrops. These assumptions may not hold in the presence of complex weather conditions, which can limit the algorithms’ effectiveness and lead to suboptimal performance in real-world scenarios. A common strategy in single-image de-raining techniques is centred around dictionary learning and sparse coding, as outlined in Kang et al. [26]. This methodology begins by decomposing the image into low-frequency and high-frequency components through a bilateral filter. Subsequently, dictionary learning is employed to distinguish rain streak components from non-rain elements within the high-frequency domain. The resulting non-rain components are then merged with the low-frequency components to yield the final de-rained image. Building upon this foundation, Chen et al. introduced depth information to enhance the de-raining process. In their work, the bilateral filter was replaced with a guided filter, which significantly improved the accuracy of distinguishing between rain streaks and non-rain components, leading to better restoration quality. Moreover, a rain removal method based on a Gaussian Mixture Model (GMM) was proposed by Li et al. [25]. This method demonstrated satisfactory results across various scenarios, effectively removing rain streaks while maintaining image integrity. However, accurately estimating the parameters of the GMM can be complex, and any inaccuracies in this estimation may result in residual raindrops remaining in the output image. Traditional single-image de-raining algorithms frequently rely on mathematical model optimisation techniques, which can lead to slow execution speeds. Additionally, these methods often leave room for improvement in terms of overall effectiveness. To address these limitations, there is a growing interest in developing more efficient algorithms that harness advances in machine learning and deep learning, aiming to achieve faster processing times while improving the quality of de-rained images. This shift towards integrating data-driven approaches with traditional physics-based models holds the potential to overcome existing challenges and enhance the performance of single-image de-raining techniques.

*Data-driven methods*: Data-driven algorithms, by design, do not depend on specific physical models, which endows them with enhanced flexibility and adaptability to a variety of weather conditions and scenarios. These algorithms can learn from extensive training datasets, allowing them to adapt to previously unseen situations and effectively tackle diverse challenges in rain removal. However, a significant drawback of data-driven approaches is their lack of interpretability regarding internal decision-making processes. This opacity can make it difficult to understand the rationale behind specific decisions made by the models. For instance, Fu et al. [30] examined the relationship between the rainy and clean detail layers, incorporating the predicted detail layer into a low-pass filtered base layer to effectively remove rain streaks. This technique aims to separate the essential image features from the rain artefacts, yet it still relies on the assumptions inherent in the data-driven framework. Similarly, Yang et al. [31] introduced a deep recurrent dilated network designed to concurrently detect and eliminate rain streaks, focusing on the temporal and spatial dimensions of the problem. This approach enhances detection capabilities but does not fully address the interpretability concerns associated with data-driven methods. Furthermore, Guo et al. [33] explored the use of Fourier priors for single image rain removal, aiming to enhance the network’s generalisation capabilities while leveraging the strong mapping abilities of convolutional neural networks (CNNs). While promising, the reliance on the Fourier Transform also presents challenges; in cases of severe degradation due to heavy rain, the phase spectrum often fails to preserve critical structural information of the original image adequately. As a result, the effectiveness of rain removal may not meet the desired standards, highlighting the need for improved methodologies that combine the strengths of data-driven approaches with more interpretable frameworks to ensure optimal performance in challenging conditions.

*Fourier Transform*: In recent years, the Fourier Transform has gained renewed interest in the field of image rain removal, owing to its ability to separate rain streaks, which predominantly occupy the high-frequency spectrum, from the lower-frequency content representing the underlying scene. This frequency-domain analysis is particularly effective for addressing the structured and repetitive nature of rain, allowing for more targeted removal techniques. Recent approaches have explored the use of Fourier Transform in combination with deep learning. For example, Li et al. (2019) proposed a model that incorporates a physics-based rain streak model with conditional adversarial learning, where the Fourier domain is utilised to guide the network in distinguishing rain streaks from vital image details. This method improves both the accuracy and generalisation of the de-raining process across different rain patterns and intensities [35]. Similarly, Yang et al. (2020) integrated Fourier analysis into a rain streak removal framework, which leverages the frequency information to improve the separation of rain from the background while reducing artefacts caused by over-smoothing [36].Furthermore, Shao et al. (2021) explored Fourier Transform directly by applying it in conjunction with deep learning models, enabling more efficient rain streak suppression. By transforming images into the frequency domain, their method effectively separates rain streaks from essential image structures, reducing artefacts and improving restoration quality [37].These recent advances demonstrate the growing role of Fourier Transform in enhancing the performance of image rain removal techniques, particularly in handling complex rain patterns and ensuring high-quality restoration.

This paper presents a novel method for single-image rain removal that effectively combines data-driven techniques with physics-based insights. Unlike our previous work [38], we have utilised Fourier Transform to efficiently separate raindrops and apply targeted enhancement processing, resulting in significantly improved efficiency. The proposed algorithm is founded on the premise that incorporating prior knowledge can significantly enhance the effectiveness of data-driven learning approaches. Recognising that both rain and noise exhibit random characteristics, we observe that the amplitude component of an image predominantly encapsulates the rain streak information, while the phase component retains the structural integrity of the background. This understanding leads to a logical framework for processing the amplitude and phase information of the rainy image separately, thereby reducing the interference from extraneous factors. To further enhance the visual quality of the rain-removed images and address the prevalent issue of detail blurriness in restored images, we introduce a multi-scale CNN. By employing convolutional kernels of varying sizes, the network is adept at extracting features at multiple scales, allowing it to capture both fine details and broader contextual information. An attention module is incorporated to adaptively assign greater weights to features that are deemed more beneficial for image restoration, effectively prioritising important details in the reconstruction process. After selecting the relevant features, they are fused to improve both the efficiency and effectiveness of the network. The architecture is well-suited to learn complex image representations, while the Fourier Transform offers a valuable perspective for understanding frequency domain information. This synergy between deep learning and Fourier analysis significantly augments the performance of rain removal algorithms. Experimental results substantiate the claim that the integration of prior knowledge is beneficial for single-image rain removal. The proposed method demonstrates its utility in various applications, particularly in fields such as autonomous navigation and smart transportation, where effective performance under rainy conditions is critical. Ultimately, this approach not only advances the state of rain removal techniques but also contributes to the broader goal of enhancing the reliability of vision-based systems in adverse weather scenarios.

## 3 The proposed de-raining algorithm

### 3.1 Framework of the proposed algorithm

The widely used rainy image model is expressed as [26]:


$$\begin{array}{ccc} I(p)=B(p)+S(p) & & \end{array}$$ (1)

where *I* represent a rainy image with rain streaks, *B* denote the underlying background layer, *S* the rain streak layer, and *p* a pixel within the image. The objective of single-image de-raining is to recover the background layer *B* from the observed rainy image *I*. This task, however, presents an ill-posed problem, particularly when the structural patterns and orientations of objects in the image closely resemble those of the rain streaks.

While Deep Convolutional Neural Networks (DCNNs) demonstrate considerable learning capabilities, achieving effective rain streak removal while simultaneously preserving intricate details remains a significant challenge. As highlighted earlier, the amplitude component of the Fourier Transform captures the majority of the rain streak information, whereas the phase component retains essential background structures and details [33]. Consequently, it is intuitive to employ the Fourier Transform to decompose the rainy image into its amplitude and phase components, allowing for separate enhancement of each.

The Fourier Transform, a powerful mathematical tool, facilitates the analysis of frequency components within an image, thereby providing insights that are not readily available in the spatial domain. By transforming the image into the frequency domain, we can effectively isolate and manipulate the amplitude and phase components. The amplitude component can be processed to suppress the rain streaks, while the phase component can be preserved to maintain the integrity of the background structure. This approach not only enhances the clarity of the resulting image but also leverages the advantages of frequency analysis, leading to a more effective solution for single-image de-raining. The following sections will provide a brief overview of the Fourier Transform, elucidating its relevance and application in our proposed method. First, perform a Fourier transform on *I* to decompose it into its real part *R* and imaginary part *M*:


$$\begin{array}{ccc} F(I_{c})(u,v)=\overset{H-1}{\underset{h=0}{\sum }}\overset{W-1}{\underset{w=0}{\sum }}I_{c}(h,w)e^{-j2\pi \left(\frac{h}{H}u+\frac{w}{W}v\right)}, & & \end{array}$$ (2)

where *c* ∈ {*R*, *G*, *B*}, *W* and *H* represent the width and height of *I*, respectively. Amplitude *A*(*x*) and $P(I_{c})$ can be expressed as:


$$\begin{array}{c} A(I_{c})(u,v)=[R^{2}(I_{c})(u,v)+M^{2}(I_{c})(u,v)]in \end{array}$$ (3)

$$\begin{array}{c} P(I_{c})(u,v)=\arctan\left[\frac{M(I_{c})(u,v)}{R(I_{c})(u,v)}\right] \end{array}$$ (4)

where *R* and *M* represent the real and imaginary part of *F*, respectively, the Fourier transformation is computed independently for each channel across all images and feature maps in our approach. It is a widely recognised fact that the phase component *P* preserves the high-level semantics of the original signal, while the amplitude component *A* contains low-level statistics. Intuitively, in a rainy image, *P* and *A* correspond to the image structures and rain streaks, respectively.

Therefore, it is advisable to enhance the amplitude and phase components separately to prevent interference from extraneous factors that may affect the overall quality of the image. To further safeguard the integrity of the phase component during processing, we incorporate an enhancement module specifically designed to elevate the latent features. This approach not only fortifies the robustness of the algorithm but also ensures that critical phase information remains intact. By focusing on these distinct components, we can achieve a more precise and effective restoration of image clarity, ultimately leading to improved performance in rain removal tasks. This dual enhancement strategy allows for a more nuanced handling of frequency domain information, enabling the algorithm to adapt more effectively to variations in the input data.

### 3.2 The Proposed Deep Convolutional Neural Network

**Fig 2:**
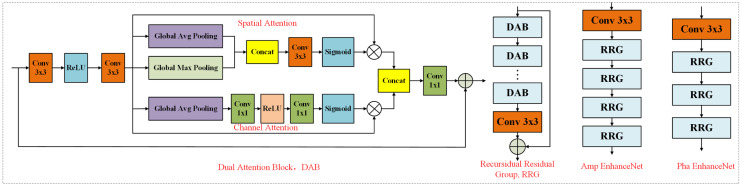
Recursive residual group (RRG), Feature Extract Net, and Rain-sreaks Extract Net. Each RRG contains multiple dual attention blocks (DAB), each DAB contains spatial and channel attention modules.

As illustrated in Figs. 1 and 2, where *P* and *A* represent the image’s structural features and rain streaks, respectively, it is beneficial to employ a DCNN to enhance these elements both separately and selectively. To efficiently leverage pertinent features, the DCNN implemented in the proposed framework is based on DeNoiseNet, as detailed in [39], which has demonstrated outstanding performance in various denoising tasks. The architecture of the recursive residual group (RRG) within DeNoiseNet is depicted in Fig 2. Each RRG consists of Dual Attention Blocks (DABs) that conduct both spatial and channel attention operations, enabling the model to focus on relevant features while disregarding less significant information. Specifically, the amplitude enhancement network is composed of four RRGs followed by a 3 × 3 convolution layer, while the phase enhancement network comprises three RRGs and one 3 × 3 convolution layer. This design allows for the suppression of irrelevant features, thereby facilitating the propagation of more informative ones throughout the network. Given that raindrops are predominantly represented within the amplitude component, the amplitude enhancement network is tasked with a more complex challenge, necessitating the inclusion of four RRGs to effectively capture and enhance the relevant details. In contrast, the phase enhancement network requires fewer RRGs, with only three, as it deals with a comparatively simpler set of features. This strategic differentiation in network design optimises the enhancement process, leading to improved performance in rain streak removal and overall image clarity.

It is well recognised that after several convolutional layers in DnCNN, high-frequency information may gradually diminish, resulting in a reduced capacity of the network to capture intricate details. This limitation can be particularly detrimental in tasks requiring a fine representation of local features. In images, details, or high-frequency information, often span multiple scales. By incorporating features from various scales, the model can effectively capture details at different levels, from broad structural elements to subtle local features. To address these challenges, we propose a Multi-Scale Feature Enhancement Branch (MSFEB) designed to further refine latent features. This innovative approach not only reduces modelling inaccuracies but also enhances the fine-grained details present in the network’s output. At each scale, the fused image is constructed through a systematic integration of features, allowing the model to leverage complementary information across different resolutions. This multi-scale strategy ensures a more comprehensive representation of the image, ultimately improving the overall performance of the network in capturing essential details while maintaining structural integrity. The MSFEB thus serves as a crucial component in enhancing the capability of deep learning models in tasks requiring high fidelity and precision in image processing.


$$\begin{array}{ll} B^{l}=N(f^{l}) & \; \end{array}$$ (5)

where *N*(⋅) denotes the network for feature fusion module, and *F*(⋅) represents the latent feature extract block as shown in Fig1.png, and $f^{l}$ is computed as


$$\begin{array}{ll} f^{l}=\{\begin{array}{ll} C(F{\left(\{I\}\right)}^{l})); & \text{if }l=1\\ C(F{\left(\{I\}\right)}^{l},N_{-1}(F{\left(\{I\}\right)}^{l-1})↑); & \text{otherwise} \end{array}\;, & \; \end{array}$$ (6)

where $N_{-1}$ denotes the network *N* without the last layer, *↑* is the up-sampling operation, and *C*(⋅) is the concatenation.

Loss functions play an important role in training the CNN from *N* pairs of images $\left\{(I,B_{T})\right\}$. The derainy image is learnt by minimizing the following loss function:


$$\begin{array}{c} L=L_{r}+L_{f}, \end{array}$$ (7)

where $L_{r}$ is defined as:


$$\begin{array}{c} L_{r}=\frac{1}{N}\underset{p}{\sum }|B_{T}-B| \end{array}$$ (8)

where $B_{T}$ is a rain-free image. $L_{r}$ can measure the differences between the rain-affected image and the clear image at a pixel-by-pixel level, thereby guiding the network’s learning process.

$L_{f}$ is defined by using the fourier transformation as:


$$\begin{array}{c} L_{f}=\frac{1}{N}\underset{p}{\sum }|A({B_{T}}_{c})(u,v)-A(B_{c})(u,v)+P({B_{T}}_{c})(u,v)-P(B_{c})(u,v)| \end{array}$$ (9)

It measures the disparity between high-frequency and low-frequency information, minimising interference from other factors and thereby ensuring a more targeted approach.

## 4 Experiments

In this section, we commence by detailing the implementation aspects of our proposed method, followed by an evaluation of its effectiveness using publicly accessible rain removal datasets. We then engage in a comparative analysis against several leading single-image rain removal algorithms, highlighting the relative strengths and weaknesses of our approach. Additionally, we conduct ablation experiments to assess the contributions of each individual module within our framework, providing insights into their respective impacts on overall performance. To facilitate a deeper understanding of the visual differences between the processed images, readers are encouraged to refer to the electronic version of this paper, where they can explore the full-sized graphics and zoom in for a more nuanced examination. The specifics are as follows:

### 4.1 Implementation Details

To evaluate the efficacy of the proposed rain removal method, we conducted comparative and analytical experiments using synthetic rain images from the Rain100L [31], Rain100H [31], and Rain1400 [40] datasets. The performance of our method was assessed using structural similarity index (SSIM) and peak signal-to-noise ratio (PSNR) metrics.

During training, we randomly cropped a 128 × 128 patch from each input image. The proposed network was trained using the suggested loss functions and the Adam optimizer with $\beta_{1}=0.9$ and $\beta_{2}=0.99$. We set the batch size to 8. The initial learning rate was set to $210^{-4}$ and subsequently decreased using a cosine annealing schedule. All experiments were implemented using PyTorch on NVIDIA GP100 GPUs. The proposed framework builds upon RRG, with the number of RRG layers in the proposed network set to four. Detailed information is illustrated in Fig 2.

### 4.2 Comparison with Existing Algorithms

All the algorithms in [31, 40–44] and the proposed algorithm include deep neural networks. Their sizes are 1.41 MB, 215 KB, 1.5 MB, 13.1 MB,34.8 MB,127.3 MB and 20.6MB, respectively. The running times of these seven different algorithms are given in Table 4. [31] and [40] were executed on the Matlab compiler. All the DNNs can be pruned in real applications.

**Fig 3:**
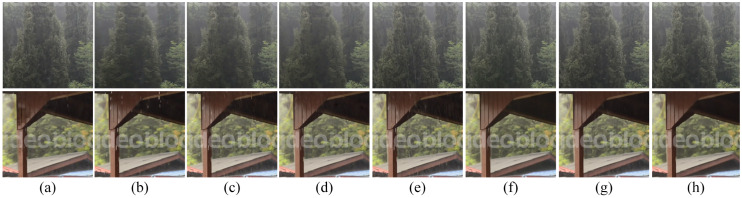
Comparison of different de-raining algorithms on real rainy images. From left to right, the restored images by [31, 40–45] and the proposed method, respectively.

**Fig 4:**
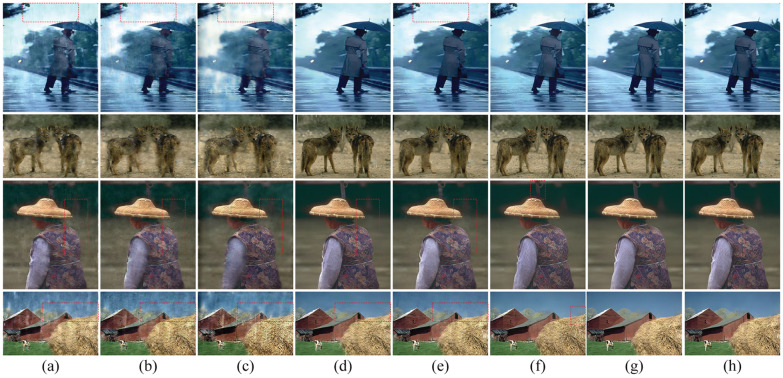
Comparison of different de-raining algorithms on synthetic rainy images. From left to right, the restored images by [31, 40–45] and the proposed method, respectively.

The proposed algorithm is compared with five state-of-the-art deraining algorithms as detailed in [31, 40–45] on a synthetic rainy dataset. These algorithms have been published in top conferences in the field of computer vision. The results for real rainy images are shown in Fig 3, while the results for simulated images are presented in Fig 4, the algorithms in [42, 43], as well as the proposed algorithm, are more effective in reducing rain streaks than those in [31, 40, 41]. Nonetheless, visible artefacts remain in the restored images produced by the algorithms in [42, 43], as indicated by the red box. The algorithm in [40] also utilises high-frequency information to learn rain streaks. However, DCNNs are not adept at learning high-frequency information, resulting in noticeable rain traces.

In addition to subjective evaluation, objective quality metrics, including SSIM and PSNR, are employed to further compare the proposed algorithm with those in [31, 40–42, 44]. The average SSIM and PSNR are presented in Tables 1–3. [45] and the proposed algorithm achieves higher PSNR and SSIM values than the other algorithms.

**Table 1:** SSIM and PSNR of different algorithms for Rain100L.

	[31]	[40]	[41]	[42]	[43]	[44]	[45]	Ours
SSIM	0.965	0.948	0.952	0.984	0.980	0.979	0.988	0.985
PSNR	34.32	32.94	32.15	38.58	38.21	36.50	40.29	38.52

**Table 2:** SSIM and PSNR of different algorithms for Rain100H.

	[31]	[40]	[41]	[42]	[43]	[44]	[45]	Ours
SSIM	0.786	0.751	0.760	0.886	0.855	0.872	0.923	0.909
PSNR	24.37	24.56	21.79	28.81	27.62	27.41	31.06	29.82

**Table 3:** SSIM and PSNR of different algorithms for Rain1400.

	[31]	[40]	[41]	[42]	[43]	[44]	[45]	Ours
SSIM	0.876	0.864	0.877	0.909	0.908	0.905	0.894	0.920
PSNR	28.37	28.19	28.21	30.59	30.60	30.32	30.71	31.45

### 4.3 Ablation Study

Key components of the proposed framework include: (1) MSFEB, (2) Multi-Scale, (3) Fourier Loss, and (4) Fourier Transform. The performances of these components are evaluated in this subsection, as illustrated in Table 5.

**Table 4:** Average running times of seven different algorithms.

	[31]	[40]	[41]	[42]	[43]	[44]	[45]	Ours
Time	8.774	1.042	0.061	0.359	0.062	0.151	0.164	0.200

**Table 5:** Ablation study on key components of the proposed framework on Rain100H (*↑*: larger is better).

Case	MSFEB	Multi Scale	Fourier Loss	Fourier Transform	SSIM (*↑*)	PSNR (*↑*)
1	N	Y	Y	Y	0.830	25.78
2	Y	N	Y	Y	0.909	28.69
3	Y	Y	N	Y	0.888	28.88
4	Y	Y	Y	N	0.908	29.79
5	Y	Y	Y	Y	0.909	29.82

**Fig 5:**
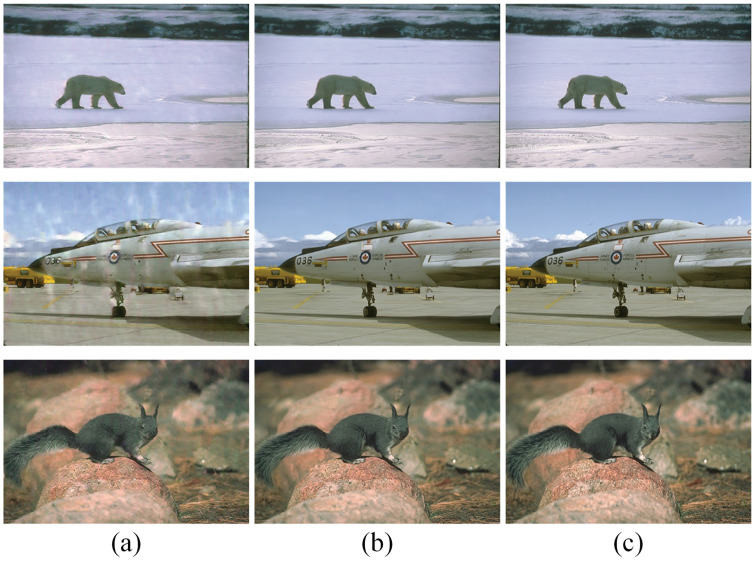
Comparison of the results of with and without MSFEB on Rain100H [31]. (a) illustrates the results without MSFEB; (b) demonstrates the images by using MSFEB. (c) presents the clean images.

**Fig 6:**
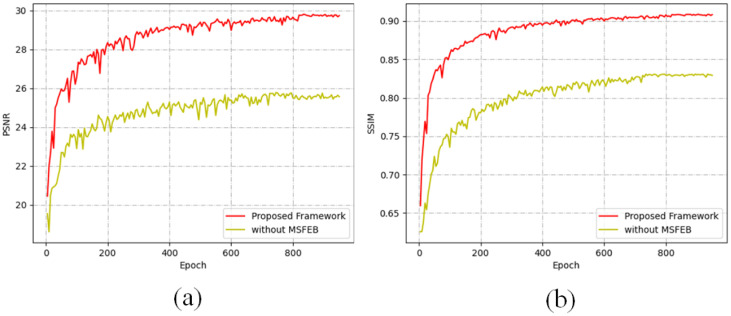
Comparison between the proposed framework with and without MSFEB for Rain100H. (a) The PSNR of two different models. (b) The SSIM of two different models. The mode with MSFEB achieves higher PSNR and SSIM.

Due to limitations in modelling accuracy, the detected raindrops may not be accurately represented, resulting in suboptimal outcomes following rain removal. To address this issue, we introduce the Multi-Scale Feature Enhance Branch (MSFEB), which is specifically designed to refine the latent features of the model. To assess the effectiveness of MSFEB, we conducted comparative experiments, evaluating the performance of the model with and without the MSFEB component in this subsection. As illustrated in Fig 5, the model incorporating MSFEB achieves a significantly higher Peak Signal-to-Noise Ratio (PSNR) compared to the version without it. Furthermore, Fig 6 demonstrates that the inclusion of MSFEB leads to markedly improved results, with enhanced clarity and detail in the processed images. In contrast, the results from the model without MSFEB reveal pronounced raindrop artefacts, highlighting the limitations of traditional approaches in accurately addressing residual noise. These findings underscore the importance of incorporating multi-scale feature enhancement to improve the overall efficacy of rain removal techniques, ultimately leading to more visually appealing and accurate image restoration.

**Fig 7:**
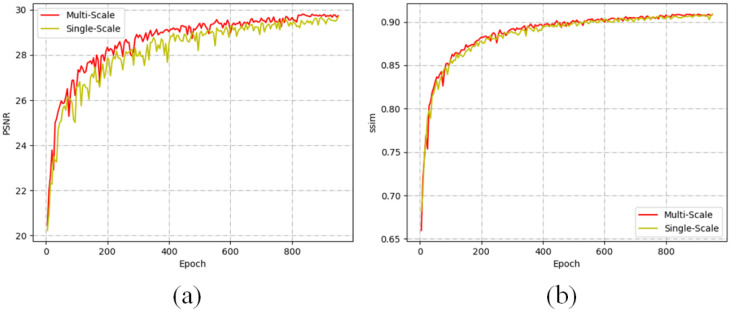
Comparison between the proposed framework with and without MSDERB for Rain100H. (a) The PSNR between multi scale and single scale. (b) The SSIM between multi scale and single scale. The mode with multi scale can achieve higher PSNR and SSIM.

To further elucidate the impact of multi-scale refinement on performance, we conducted a comparative analysis with a baseline method that does not utilise multi-scale techniques but is implemented in a manner consistent with our approach. The evaluation was performed on the Rain100H dataset, a widely recognised benchmark for assessing rain removal algorithms. As illustrated in Fig 7, the dual-scale method consistently achieves higher and more stable PSNR values compared to the single-scale approach. This finding underscores the effectiveness of our multi-scale refinement strategy, which not only enhances overall image quality but also contributes to greater stability in performance across varying conditions. Such improvements highlight the significant advantages of incorporating multi-scale features in the de-raining process, reinforcing the robustness of our proposed methodology.

The Mean Squared Error (MSE) loss function serves as a point-to-point metric that quantifies the average squared difference between predicted and actual values. However, its sensitivity to outliers and the absence of translational invariance can lead to a loss of detail in specific contexts. In particular, image processing tasks that require meticulous detail preservation may suffer from the excessive smoothing or blurring that MSE can induce. To address these shortcomings and enhance detail retention in de-rained images, this paper introduces the Fourier Loss function.

**Fig 8:**
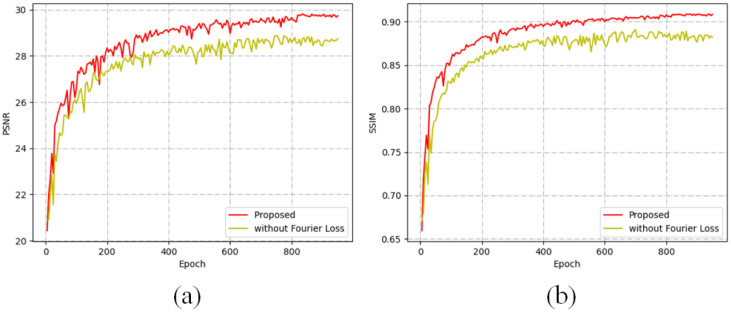
Comparison between the proposed framework with and without Fourier Loss Function for Rain100H. (a) The PSNR between the model with and without Fourier Loss Function. (b) The SSIM between the model with and without Fourier Loss Function. The mode with Fourier Loss Function achieves higher PSNR and SSIM.

**Fig 9:**
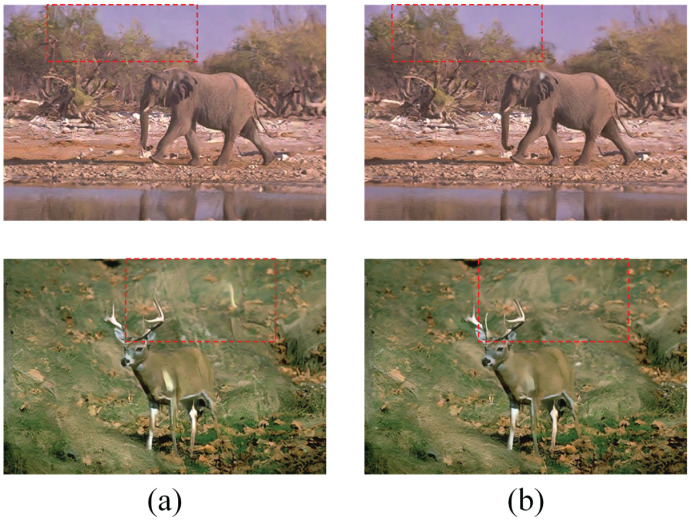
Comparison the results with and without Fourier Loss Function for Rain100H. (a) are the results of model without Fourier Loss Function. (b) are the results of model with Fourier Loss Function.

This innovative loss function exploits the distinct characteristics of the frequency domain, wherein the amplitude component encapsulates the majority of high-frequency information, while the phase component ensures the consistency of low-frequency information. By maintaining the coherence of both amplitude and phase in the output image, our approach effectively mitigates the drawbacks associated with MSE. As illustrated in Fig 8, models employing the Fourier Loss function demonstrate significantly improved Peak Signal-to-Noise Ratio (PSNR) and Structural Similarity Index (SSIM) scores, indicating superior performance in detail preservation. To further validate the efficacy of the Fourier Loss function, we conducted subjective experiments, the results of which are depicted in Fig 9. The findings, highlighted in the red box, reveal that outputs from the model without the Fourier Loss function exhibit prominent artifacts and a marked lack of clarity. In contrast, the model incorporating the Fourier Loss function successfully retains finer details, providing clearer and more visually appealing results. These observations underscore the advantages of using the Fourier Loss function in image de-raining applications, as it fosters enhanced detail preservation and mitigates the issues typically associated with traditional loss functions like MSE.

**Fig 10:**
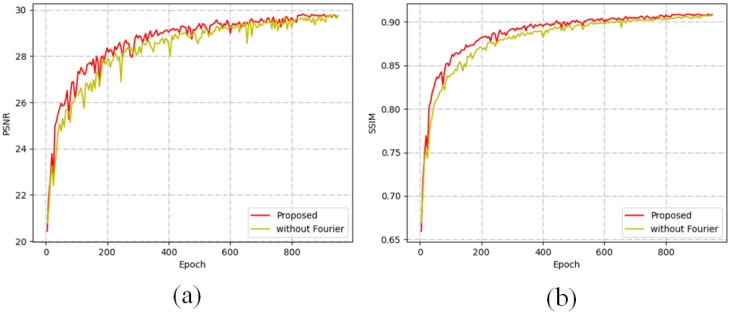
Comparison between the proposed framework with and without Fourier for Rain100H. (a) The PSNR between the model with and without Fourier. (b) The SSIM between the model with and without Fourier. The mode with Fourier can achieve higher PSNR and SSIM.

It has been well established that the amplitude component of an image contains the majority of the information related to rain streaks, while the phase component preserves the structural characteristics of the background. By decomposing the image into amplitude and phase maps and selectively enhancing these components within the feature domain, we can improve the efficiency of network training and reduce interference from extraneous factors. To assess the significance of the Fourier Transform in our proposed framework, we conducted an experiment in which the Fourier Transform was omitted, while all other modules remained unchanged. As illustrated in Fig 10, the framework that incorporates the Fourier Transform converges more rapidly and exhibits a more stable training curve compared to the model without this transform. This indicates that the inclusion of the Fourier Transform not only accelerates the training process but also enhances the overall robustness of the model, underscoring its critical role in the image de-raining process. Such findings suggest that integrating frequency domain information can substantially contribute to the effectiveness of deep learning architectures in complex image restoration tasks.

## 5 Conclusion remarks and discussions

This paper presents a novel algorithm for single-image de-raining that effectively integrates both data-driven and model-based methodologies. We introduce the use of Fourier prior knowledge for image de-raining, demonstrating its ability to enhance both generalisability and performance. It is well established that the amplitude component primarily captures rain streak information, while the phase component retains structural similarities to the background. By applying the Fourier Transform, raindrop images are decomposed into phase and amplitude maps, which undergo targeted enhancement processes to improve training efficiency. Given that raindrops also encompass low-frequency information, further refinement of the recovered latent features is crucial. To this end, we employ a multi-scale attention mechanism within a network to enhance de-raining performance while preserving intricate details. Experimental results indicate that the proposed algorithm significantly outperforms several existing single-image de-raining methods.

For other low-level visual processing tasks, such as dehazing and illumination enhancement, integrating prior knowledge into data-driven methodologies can also prove beneficial. Beyond this, it is crucial to blend model-based approaches with data-driven techniques for effective low-level visual processing. The proposed algorithm may also be adapted to enhance the study of autonomous navigation under rainy conditions. These aspects will be explored further in our future research.
